# A Fusion Peptide in the Spike Protein of MERS Coronavirus

**DOI:** 10.3390/v11090825

**Published:** 2019-09-05

**Authors:** Entedar A. J. Alsaadi, Benjamin W. Neuman, Ian M. Jones

**Affiliations:** 1 School of Biological Sciences, University of Reading, Reading RG6 6AJ, UK; 2 Biology Department, CASE, Texas A&M University, Texarkana, TX 75503, USA

**Keywords:** coronavirus, MERS, spike protein, peptide, membrane, fusion assay

## Abstract

Coronaviruses represent current and emerging threats for many species, including humans. Middle East respiratory syndrome-related coronavirus (MERS-CoV) is responsible for sporadic infections in mostly Middle Eastern countries, with occasional transfer elsewhere. A key step in the MERS-CoV replication cycle is the fusion of the virus and host cell membranes mediated by the virus spike protein, S. The location of the fusion peptide within the MERS S protein has not been precisely mapped. We used isolated peptides and giant unilamellar vesicles (GUV) to demonstrate membrane binding for a peptide located near the N-terminus of the S2 domain. Key residues required for activity were mapped by amino acid replacement and their relevance in vitro tested by their introduction into recombinant MERS S protein expressed in mammalian cells. Mutations preventing membrane binding in vitro also abolished S-mediated syncytium formation consistent with the identified peptide acting as the fusion peptide for the S protein of MERS-CoV.

## 1. Introduction

Coronaviruses, positive strand non-segmented RNA viruses with the largest RNA genomes known, infect a wide range of hosts, including many mammalian species, and cause a range of diseases, including respiratory, gastrointestinal, hepatic and neurological diseases [1]. Viruses of zoonotic potential are found within the coronaviruses, as exemplified by severe acute respiratory syndrome-related coronavirus (SARS-CoV), which emerged in Southern China in 2003 [2], and Middle East respiratory syndrome-related coronavirus (MERS-CoV), which appeared in Saudi Arabia in 2012 [3]. In both cases the infection of humans is thought to have arisen by contact with an intermediate host which in turn acquired the virus from the original reservoir, presumed to be bats [4,5]. The basis of cross-species infection lies primarily in the ability of the virus major surface spike protein, S, to bind to cell surface receptors and initiate infection. Coronaviruses use a variety of receptors ranging from sugars to extended cell surface proteins (reviewed in [6]) and the entry receptor for MERS infection has been identified as dipeptidyl peptidase-4 (DPP4), found on a variety of cell types including epithelial cells of the respiratory tract [7,8]. More recently, sialic acid has been shown to be an additional low-affinity attachment receptor whose binding might precede that of DPP4, suggesting that its distribution may also contribute to virus tropism [9].

Receptor binding by the S protein occurs via the N-terminal S1 domain [10] while the conformational change that follows is driven by the S2 domain, leading to the fusion of the viral and cellular membranes and the release of the viral genome into the cytoplasm [11]. Separation of the S1 and S2 domains is mediated by host cell proteases including members of the cathepsin family and transmembrane protease serine 2 (TMPRSS2) [12]. The S2 protein comprises the fusion peptide, two conserved heptad repeats (HR) (distant in the primary amino acid sequence but adjacent in the post-fusion conformation), a transmembrane domain and a short intracellular tail [11]. The structural features of S, including the fusion peptide, are essential for spike function and have been suggested as targets for therapeutic intervention [13]. Fusion peptides have been generally described as sequences of 15–25 apolar amino acids which locate to and order membranes on binding to enable the fusion process to begin [14]. The boundaries of coronavirus fusion peptides have yet to be precisely mapped, but the consensus is that the S fusion peptide lies in the N-terminus of the S2 domain, just downstream of the S1/S2 boundary [15]. For SARS-CoV S, mutagenesis, structural and lipid mixing studies have suggested a motif SFIEDLLFNKVTLADAGF which is conserved across the coronavirus family and within which the core sequence IEDLLF demonstrates only infrequent and conservative replacements [16,17]. Most recently, this putative SARS fusion peptide has been shown to induce Ca^2+^-dependent membrane ordering consistent with its role during virus–host membrane fusion [18].

Here we investigate the putative fusion peptide of MERS-CoV S and demonstrate membrane binding and deformation by incubation with giant unilamellar vesicles (GUVs). Alanine scanning mutagenesis of the core sequence coupled with further GUV binding was used to identity residues critical for activity. Finally, a syncytium assay using recombinant MERS S protein was established and the critical residues were tested for their role in syncytium-forming activity.

## 2. Materials and Methods

### 2.1. Peptide Synthesis

All peptides were based on the sequence AHX00731.1 (a camel isolate identical to a number of other deposited sequences) and were provided by Cambridge Research Biochemicals, UK. Peptides were provided as lyophilized products with a mean purity of ≥70%. Peptide stock solutions were prepared in a buffer consisting of 0.1 mM sucrose, 0.1 mM glucose, 10 mM DTT, 0.5% (*v*/*v*) DMSO.

### 2.2. Giant Unilamellar Vesicles (GUVs)

GUVs were generated by electroformation using a Vesicle Prep Pro (Nanion Technologies GmbH, Munich, Germany) using a mix of 5 mM 1,2-dipalmitoyl-*sn*-glycero-3-phosphocholine (DPPC), 4 mM egg sphingomyelin and 0.5 mol% cholesterol, which were first dissolved in chloroform. To visualize the GUVs, 0.5 mol% of naphtha [2,3-alpha] pyrene (Tokyo Chemical Industry UK Ltd., Oxford, UK) was added. Lipids were mixed in an amber vial to a final total lipid concentration of 9 mM before preparation by electroformation. Briefly, 20 µL of lipid stock was spread on the conductive side of an indium tin oxide slide (ITO slide). After the evaporation of the solvent, the slide was put into a vacuum desiccator for 1 h to remove any remaining trace of the solvent. A 28 mm O-ring was coated on one side with silicon grease and placed around the dried lipid film to prevent leakage of the rehydration solution. The lipid film was hydrated with 750 µL of 0.1 mM sucrose solution in deionized water. Then the second ITO slide was placed on top of the O-ring with the conductive sides facing each other. A tension of 3 V peak to peak and a frequency of 5 Hz was applied to the ITO slides over a period of 2 h at 50 °C [19]. GUVs were imaged using an EVOS-FL digital fluorescence microscope (ThermoFisher Scientific, Waltham, MA, USA).

### 2.3. GUV Deformation Assay

Following electroformation, the chamber was disassembled and the sucrose solution removed to leave the GUVs attached to one ITO slide. The diluted peptide was added immediately to the desired concentration and the field was imaged at 0, 1, 2 and 5 min post peptide addition. ImageJ was used to measure the shape and the relative size of the GUVs. Experiments were performed in triplicate and the average and standard deviation were calculated. The M2-influenza peptide was chosen as a positive control, as it is a well-characterized and highly conserved amphipathic helix sufficient for budding into GUVs and for the formation of large luminal vesicles (LUVs) [20]. Buffer-only controls were treated in the same way. GUVs–peptide incubation was conducted at room temperature.

### 2.4. Statistical Analysis

Statistical significance was calculated using SPSS version 22, using a Linear Mixed Model (LMM) (*p* < 0.05). Results were expressed as the mean ± SEM. Figures were generated using GraphPad Prism 7.0b software.

### 2.5. Cell Culture

All cells were cultured and maintained in Dulbecco’s modified Eagle medium (DMEM) (Sigma Aldrich, St. Louis, MO, USA) supplemented with 10% fetal bovine serum (FBS) (GE Healthcare, Chicago, IL, USA) and 0.2% penicillin-streptomycin solution (Gibco/Invitrogen, Carlsbad, CA, USA) at 37 °C with 5% CO_2_.

### 2.6. Constructs and Mutagenesis

The MERS-CoV spike protein coding region was synthesized de novo (Integrated DNA Technologies, Coralville, IA, USA) and inserted between the NcoI and XhoI sites of vector pTriEx 1.1 (Merck Millipore, Burlington, MA, USA). Mutants were synthesized and cloned similarly. All constructs were confirmed by DNA sequencing prior to use.

### 2.7. Syncytium Formation

A total of 1.25 × 10^5^ Lenti-X 293T cells (Takara Bio Inc., Tokyo, Japan) were seeded on a glass coverslip in a 12 well plate and incubated for 24 h at 37 °C. Cells were transfected with 1 μg of plasmid DNA using Lipofectamine 3000 (Invitrogen) for 24 h at 37 °C. Prior to the assay, S cleavage was ensured by treatment with 2 μg/mL of trypsin in Opti-MEM (Sigma Aldrich) for 30 min at 37 °C as previously described [17]. The medium was removed and the cells rinsed once with PBS adjusted to pH 5.0 (with citric acid) followed by incubation for 5 min in the same buffer at 22 °C. The PBS was replaced with complete DMEM for 1 h at 37 °C and the monolayer fixed and permeabilized using Bioscience^TM^ Intracellular Fixation and Permeabilization Buffer (ThermoFisher, Waltham, MA, USA) prior to immunofluorescence staining.

### 2.8. Immunofluorescence

Permeabilized cells were incubated with primary antibody, either anti-MERS-CoV Spike (D12) monoclonal antibody (Ab00696, Absolute Antibody, Redcar, UK, at 1:200 dilution) or anti DPP4 (Anti-CD26 antibody ab119346, Abcam, UK at 1:20 dilution), for 1 h, then washed and incubated with a 1:200 dilution of Alexa Fluor 488 goat anti-mouse antibody (ThermoFisher, Waltham, MA, USA) for 1 h. Following the final washing, the stained cells were counterstained with Slowfade^TM^ Gold antifade reagent with DAPI (Invitrogen) and visualized using an EVOS-FL digital fluorescence microscope (ThermoFisher, Waltham, MA, USA).

## 3. Results

### 3.1. Identification of the Putative MERS-CoV Fusion Peptide

**Figure 1:**
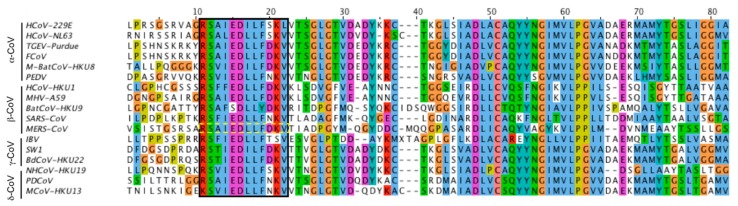
Multiple sequence alignment of coronavirus spike protein S2 subunit. Alignment of 17 coronavirus partial S2 sequences representing four genera of coronavirus. The isolates are: α-Alphacoronavirus (α-CoV) HCoV-229E (ABB90529.1); HCoV-NL63 (YP_003767.1); TGEV (ABG89335.1); FCoV (YP_004070194.1 AFH58021); M-Bat CoV-HKU8 (YP_001718612.1); and PEDV (NP_598310.1). Betacoronavirus (β-CoV) HCoV-HKU1 (ADN03339.1); MHV-A59 (NP_045300.1); BatCoV-HKU9 (YP_001039971.1); SARS-CoV (NP_828851.1); and MERS-CoV (AHX00731.1). Gammacoronavirus (γ-CoV) IBV (ADP06471.2); SW1 CoV (YP_001876437.1); and BdCoV-HKU22 (AHB63508.1). Deltacoronavirus (δ-CoV) NHCoV-HKU19 (AFD29226.1); PDCoV (AFD29187.1); and MCoV-HKU13 (YP_002308506.1). The identified Middle East respiratory syndrome (MERS) putative fusion peptide is boxed.

Analysis of the MERS spike S2 domain (residues 761–1353) with AmphipaSeek [21] identified an amino acid sequence RSARSAIEDLLFDKV, residues 884–898, with properties consistent with a putative fusion peptide that was highly conserved among 17 different spike sequences constituting the α, β, δ and γ coronavirus genera (Figure 1). The identified sequence, which is related to the SARS-CoV putative fusion peptide [18] is not located at the N-terminus of HR1 as suggested in some S protein cleavage maps (e.g., [13]), but immediately follows the second (S2′) cleavage site originally mapped in SARS-CoV S and later in MERS-CoV S [22,23]. Given its high degree of conservation and its location at a critical biologically relevant junction, the 15 residue wild type peptide was synthesized for in vitro activity assessment along with five variants in which selected hydrophobic residues previously implicated in the SARS fusion peptide were exchanged for alanine to probe those positions that were essential for activity (Table 1).

### 3.2. Effect of MERS-CoV Putative Fusion Peptide on Shape and Size of GUVs

**Figure 2:**
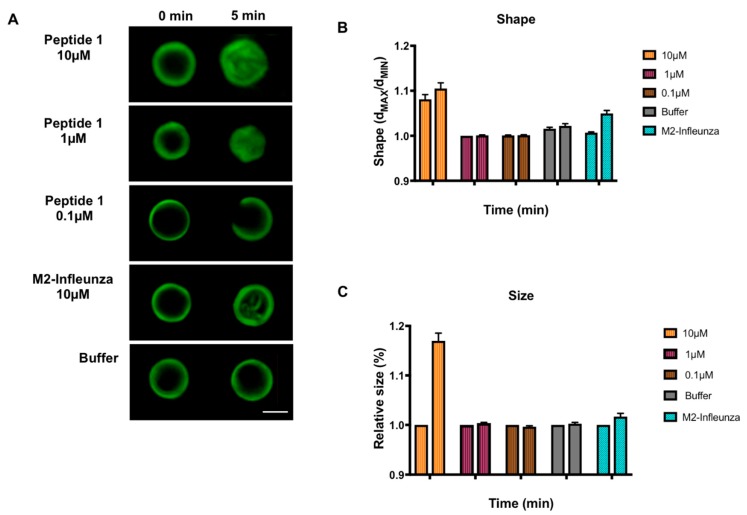
Effect of wild type MERS-CoV putative fusion peptide on the shape and size of giant unilamellar vesicles (GUVs). (**A**) Fluorescent images of electroformed GUVs treated with 10, 1 and 0.1 µM peptide 1, or 10 µM M2-Influenza and imaged at 0 and 5 min. Scale bar at bottom right indicates 5 µm. (**B**) GUV shape. (**C**) Relative GUV size. Statistical significance was shown for peptide 1 at 10 µM compared to buffer only at all time points for both size and shape of the GUV (*p* < 0.01; Linear Mixed Model). Error bars shown are the mean ± SEM. The scale bar indicates 20 µm. Each colored group of two columns left to right represents the data for a single peptide tested at each time point, 0 and 5 min. Some error bars are too small to be observed.

To address its potential as a fusion peptide, the 15 residue wild type (WT) peptide was included in the reconstitution of GUV membranes and the effect on size and morphology was measured. GUVs, composed of 5 mM DPPC, 4 mM egg SM and 0.5 mol% cholesterol, were reconstituted with the peptides at concentrations of 10, 1 and 0.1 µM. All samples also contained 0.5% naphthopyrene to allow observation by fluorescence microscopy. Their shape was measured as the ratio between the longest and shortest radii while the relative size of the GUVs was estimated by Ramanujan’s first approximation, taking an average of 40 GUVs per experiment for three separate experiments, with vesicles only and the M2-Influenza peptide acting as controls (Figure 2). The WT peptide was shown to change the shape and size of the GUV membrane, leading to extensive deformation at 10 µM. At the lower peptide concentration of 1 µM, the absolute size and shape did not vary despite a change in GUV appearance with time. Meanwhile, at 0.1 µM the putative fusion peptide caused membrane permeability due to pore formation observed as loss of fluorescence, leading to a cuplike shape, as described elsewhere [24]. Control GUVs treated with buffer only showed no deformation, while the M2-Influenza peptide at 10 µM concentration led to GUVs budding and forming large luminal vesicles, as described in [20], thus validating the assay.

**Figure 3:**
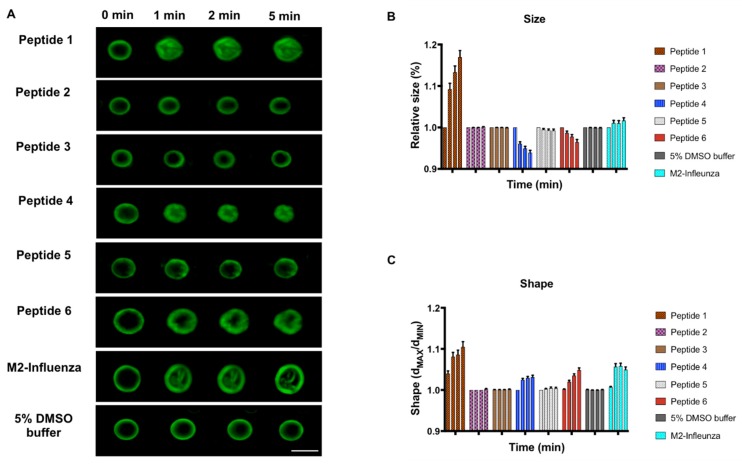
Comparison of wild type and mutant MERS-CoV putative fusion peptides on GUV shape and size. (**A**) Fluorescent images of electroformed GUVs treated with 10 µM peptides 1–6, M2-Influenza or buffer alone, and imaged at 0, 1, 2 and 5 min after peptide addition. Scale bar at lower right indicates 5 µM. (**B**) GUV shape. (**C**) GUV size. Statistical significance was shown for peptides 1, 4 and 6 at all the time points for both size and shape of the GUV (*p* < 0.01; Linear Mixed Model). Peptides 2, 3 and 5 did not show a statistically significant change in shape or size when compared to buffer only. All error bars shown are the mean ± SEM (Linear Mixed Model; *p* < 0.01). The scale bar indicates 20 µm. Each colored group of four columns, left to right, represents the data for a single peptide tested at each time point 0, 1, 2 and 5 min. Some error bars are too small to be observed.

To address the key residues within the putative fusion peptide, a series of five additional peptides (Table 1) were included with the reconstituted GUVs, all at 10 µM, and their shape and size were measured as before. While peptides 4 (L894A) and 6 (V898A) continued to show some GUV membrane deformation, albeit with different effects compared to the WT sequence, peptides 2 (I890A), 3 (L893A) and 5 (F895A) did not lead to appreciable deformation in shape or size over the course of the observation, suggesting that these residues are critical for activity (Figure 3). Interestingly, when aligned with the limited S sequences selected for the original bioinformatics screen, positions Ile 890 and Leu 893 are invariant, while Phe 895 undergoes one highly conservative change to Tyr in the S protein of Bat HKU9 CoV (cf. Figure 1).

### 3.3. Role of Identified Peptides in Syncytium Formation

**Figure 4:**
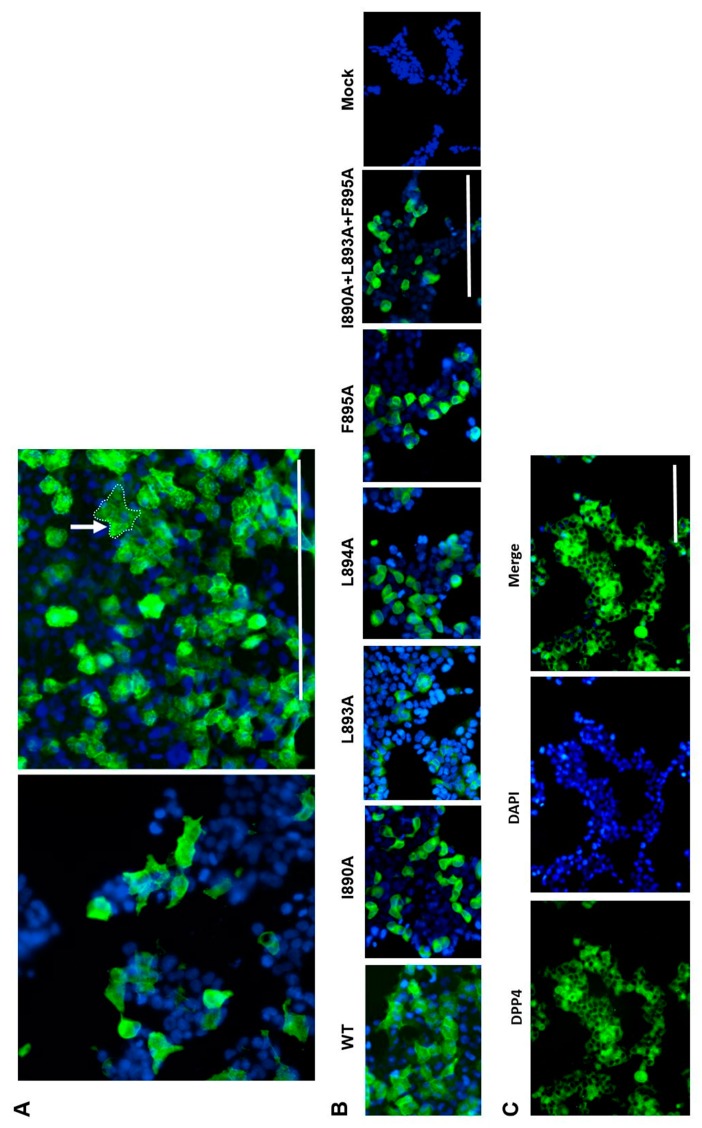
MERS S expression and syncytium formation. Lenti-X 239T cells were transfected with vectors encoding WT MERS-CoV S or the alanine mutants described in the text. (**A**) Cells transfected with the WT S were treated with 2 µg trypsin/mL for 30 min at 37 °C prior to a 5 min acid pulse. Following recovery in complete Dulbecco’s modified Eagle medium (DMEM) for 1 h at 37 °C transfected non-treated (4A left) and transfected + acid treated (4A right) cells were processed for immunofluorescence and visualized as described. A syncytium (arrow) is shown with boundaries highlighted with a dotted line. All images were recorded using the 20× objective. (**B**) WT and alanine scanning mutants of S as indicated were transfected and acid pulsed before being processed for immunofluorescence as before. (**C**) Confirmation of DPP4 receptor expression revealed by staining Lenti-X-239T cells with a primary anti-CD26 MAb followed by an anti-mouse Fluor 488 conjugate. Nuclei were counterstained with DAPI (blue). The scale bars are 200 µm.

To confirm a role for the putative fusion peptide in a biological system, a syncytium assay based on the expression of complete S protein was established, similar to that described for the avian coronavirus IBV [25]. The S coding region was expressed from the CAG promoter present in vector pTriEx1.1 following the transfection of Lenti-X 293T cells and was detected with a monoclonal antibody specific for S. The presence of the receptor DPP4 was confirmed by staining with a DPP4 monoclonal antibody (Figure 4C). When transfected cells were cultured in the presence of trypsin, to ensure the maturation of the spike protein when endogenous cleavage is inefficient [23], and subject to an acid pulse followed by a period of recovery, profuse syncytia characterized by merged cells (indicated) with diffuse S staining extending into non-transfected adjacent cells and very few individual cells were observed, consistent with S-mediated fusion (Figure 4A). To assess the role of the residues identified in the peptide–GUV binding assays in syncytium formation, each mutation was introduced into the S sequence and the assays repeated with syncytium forming ability screened by acid pulse as before. In all cases, while the expression of MERS-CoV S with mutations at I890A, L893A, L894A and F895A did not alter the expression of the protein as judged by the intensity of S antibody staining, cell-to-cell fusion was not apparent following the post-transfection treatment. Fields typically contained single cells with S staining in the cytosol and at the cell periphery but not extending into non-transfected adjacent cells (Figure 4B). These data are correlated with the membrane deformation activity of peptides that included the same mutations and confirm the I_890_L_893_F_895_ core identified by GUV binding as essential for activity as well as critical for cell fusion.

## 4. Discussion

The fusion protein S of coronavirus is essential for virus infectivity, while antisera that block either receptor binding or fusion activity have a protective effect [26,27,28]. In addition, S is a target for therapeutic intervention via peptides that compete with the fusion reaction or the cleavage between S1 and S2 [13]. Here, alignments of the spike protein of MERS with several other coronavirus spike proteins revealed a conserved sequence just downstream of the S2′ cleavage site with homology to a sequence previously reported to contain the fusion peptide of SARS S. Studies with the relevant peptide showed that it increased the size and deformed the shape of GUVs, consistent with the partition of the peptide into the external leaflet of the lipid membrane, as has been reported for peptides derived from other amphipathic membrane-inserting proteins such as Melittin [24] or Sar1p [29].

Single amino acid substitution experiments revealed a key role in GUV deformation for hydrophobic amino acids isoleucine, leucine and phenylalanine located in the central region of the putative fusion peptide, and the same residues were found to be critical for syncytium-forming ability when incorporated into the full-length MERS S protein expressed in DPP4-expressing mammalian cells. Together, the experimental data for the MERS sequence coupled with the similarity of the defined sequence with the data obtained for SARS suggest that the sequence RSARSAIEDLLFDKV and particularly the core sequence IEDLLF constitute the fusion peptide for MERS coronavirus S protein.

## Figures and Tables

**Table 1:** MERS-CoV peptides. Mutated residues are underlined in boldface.

No.	Designation	Residues	Sequence
1	Peptide 1 (wild type)	884–898	RSARSAIEDLLFDKV
2	Peptide 2	I890A	RSARSA**A**EDLLFDKV
3	Peptide 3	L893A	RSARSAIED**A**LFDKV
4	Peptide 4	L894A	RSARSAIEDL**A**FDKV
5	Peptide 5	F895A	RSARSAIEDLL**A**DKV
6	Peptide 6	V898A	RSARSAIEDLLFDK**A**
